# Salivary gland macrophages in health and disease: heterogeneity, niche crosstalk, and therapeutic avenues

**DOI:** 10.3389/fimmu.2025.1688738

**Published:** 2025-10-14

**Authors:** Xinglei Li, Yan Feng, Huixin Xue, Xinxin Ni

**Affiliations:** Department of Stomatology, the First Affiliated Hospital, School of Medicine, Zhejiang University, Hangzhou, China

**Keywords:** salivary glands, macrophages, macrophage heterogeneity, niche crosstalk, sexual dimorphism, Sjögren’s disease, therapeutic strategies, saliva biomarkers

## Abstract

Salivary glands (SGs) produce saliva essential for digestion, mucosal immunity, and as a source of non-invasive biomarkers. The health and pathology of SGs hinge upon a dynamic equilibrium between two core macrophage (Mφ) populations: long-lived, tissue-resident 'guardians' that maintain homeostasis, and short-lived, monocyte-derived 'sentinels' that mount rapid responses. This review posits an integrative framework wherein the disruption of this balance is a central pathogenic hub across diverse SG disorders, from autoimmunity to radiation-induced injury. We reconcile long-standing controversies over Mφ origins by proposing a 'developmental transition model' and highlighting the pivotal discovery of sexual dimorphism—a fundamental difference in Mφ maintenance between males and females. Drawing on advances in single-cell omics and spatial imaging, we redefine SG Mφ heterogeneity far beyond the M1/M2 paradigm, recasting them as critical 'communication hubs' within the neuro-epithelial-immune network. This new perspective inspires a paradigm shift in translational priorities, moving from broad immunosuppression towards precisely 'restoring ecological balance'. We prioritize therapeutic strategies such as selectively protecting guardian Mφs via transient Hedgehog pathway activation, and promoting regeneration through transplantation of engineered effective-mononuclear cells (E-MNCs), a strategy with emerging clinical validation. Furthermore, we survey the frontier of targeting tumour-associated Mφs and underscore the immense potential of developing non-invasive salivary biomarkers for immune monitoring, charting a course to bridge critical translational gaps.

## 1 Introduction

Salivary glands (SGs) are essential for oral and systemic health, producing saliva that is vital for digestion and immunity and serves as a rich medium for non-invasive biomarkers (1, 2). The major SGs—comprising the parotid, submandibular, and sublingual glands—exhibit remarkable structural and functional conservation across mammals, including humans, swine and rodent models (3). However, SG dysfunction, often presenting as xerostomia (dry mouth), is a globally prevalent condition affecting a quarter of the population. Driven by diverse etiologies such as Sjögren's disease (SjD), head and neck radiotherapy, and infections, this dysfunction severely compromises salivation, leading to a cascade of oral diseases and a profound decline in quality of life (4–11). With current therapies limited to palliative care that fails to restore glandular function, there is a critical unmet need for a deeper understanding of the molecular mechanisms governing SG homeostasis to develop effective, restorative treatments (12, 13).

**Figure 1:**
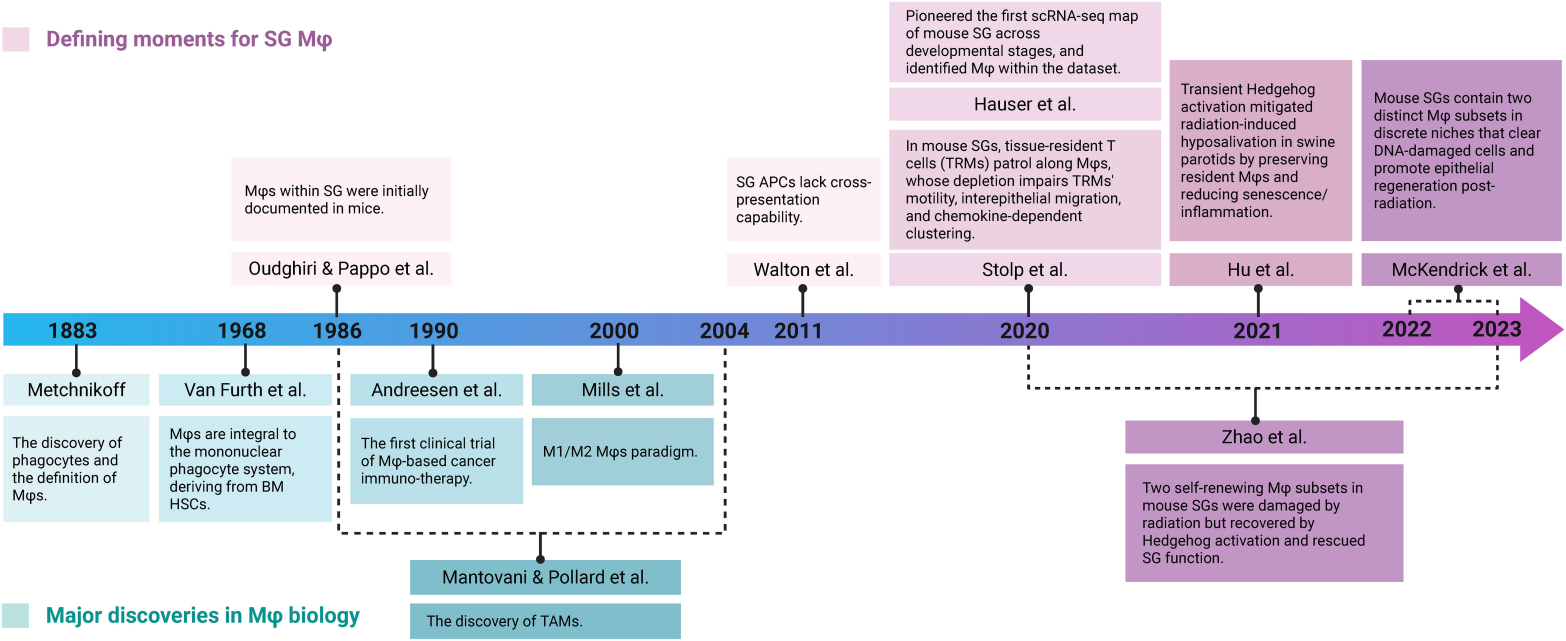
Timeline of key discoveries in Mφ biology and SG Mφ research. Key findings are categorized into foundational advances in broader Mφ biology (teal) and research specifically focused on SG Mφs (purple). Early foundational work on broader Mφ biology (e.g., Metchnikoff's discovery in 1883, the M1/M2 paradigm in 2000, and TAM identification) provided context. Dedicated SG Mφs research began in 1986 with their identification in mouse SG. More recent studies highlight SG Mφs' unique characteristics and functions in homeostasis (Walton et al., 2011, Stolp et al., 2020, Hauser et al., 2020) and tissue regeneration after injury (McKendrick et al., 2022-2023, Zhao et al., 2020-2023). Crucially, the validation of these Mφ-mediated pro-regenerative functions in a swine model enhanced the translational relevance, bridging the gap between rodent studies and human SG Mφ biology (Hu et al., 2021). Image created with BioRender.com.

Within the intricate cellular landscape of the SGs, macrophages (Mφs) are emerging as pivotal regulators of both immunity and tissue function (13–16). Historically viewed as simple phagocytes, Mφs are now recognized for their remarkable heterogeneity and plasticity, allowing them to perform tissue-specific roles in development, homeostasis, and repair (17). In SGs, Mφs are the dominant innate immune cell type, accounting for 90% of CD45^+^ MHC II^+^ antigen-presenting cells, yet their specific functions have remained surprisingly under-explored since their initial description in 1986 (18–21) (**Figure 1**). Their unique location and critical roles in both immune and non-immune functions (e.g., secretion) make SG Mφs a compelling model to decipher universal regulatory principles of tissue-resident Mφs in maintaining homeostasis and promoting disease across various exocrine glands and other organ systems. Despite early descriptions of their presence and general involvement in inflammation (16), a comprehensive understanding of the heterogeneity, niche-specific adaptations, and precise functional contributions of distinct SG Mφ populations in health and disease has remained surprisingly elusive. This knowledge gap stands in sharp contrast to the rapidly advancing understanding of Mφ biology in other organs, highlighting SG Mφs as a compelling and underexplored model for dissecting fundamental principles of tissue-resident Mφ function.

Recent technological breakthroughs, particularly single-cell omics and high-resolution imaging, are revolutionizing the field, enabling an unprecedented view of the SG Mφ landscape (16, 22–30). These tools allow researchers to move beyond historical classifications to define distinct Mφ subpopulations, map their spatial organization, characterize their activation states, and decipher their intricate crosstalk with epithelial, stromal, and other immune cells.

This review synthesizes the rapidly evolving understanding of SG Mφs, detailing their developmental origins, heterogeneity, and the critical role of niche-driven intercellular communication. We provide mechanistic insights into how these versatile cells contribute to SG physiology and the pathogenesis of prevalent disorders, including SjD and radiation-induced injury. Ultimately, by highlighting cutting-edge research and identifying key unanswered questions, this review aims to stimulate a paradigm shift in our understanding of gland biology and complex neuro-epithelial-immune interactions, positioning SG Mφs as vital therapeutic targets and their secreted factors in saliva as powerful biomarkers for both oral and systemic health.

## 2 Origins and heterogeneity of SG Mφs

### 2.1 Developmental origins and maintenance

**Figure 2:**
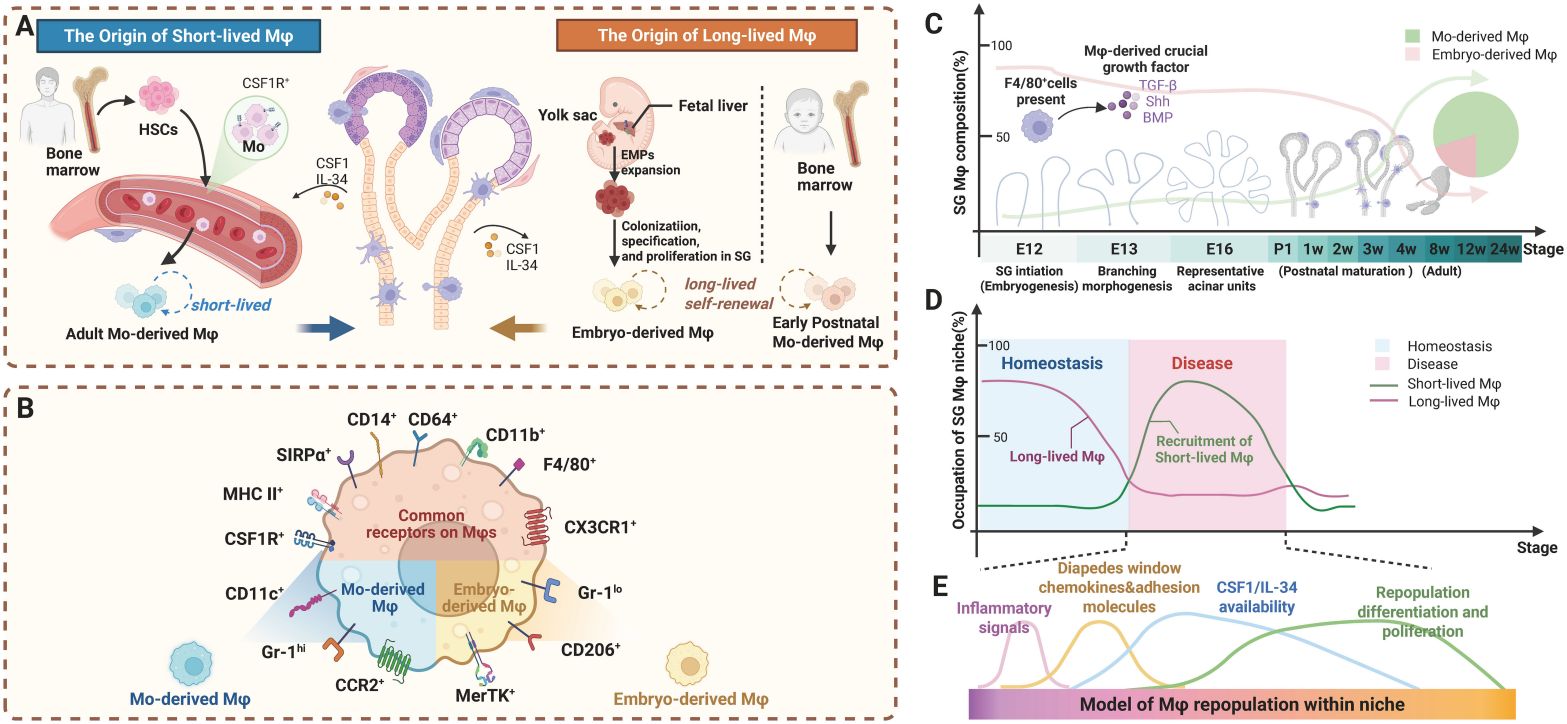
Ontogeny and dynamic remodeling of SG Mφ subsets: long-lived vs. short-lived populations during development and disease. **(A)** Distinct origins and longevity of SG Mφs. The long-lived Mφ pool is seeded by both embryonic progenitors and early postnatal monocytes, together forming a self-sustaining population. Conversely, the short-lived Mφ subset originates from adult BM-derived monocytes **(B)** Surface marker profiles differentiate Mφ subsets. Mo-derived Mφs are Gr-1^hi^, CD14^+^, CCR2^+^, and CX3CR1^lo^. Embryo-derived Mφs are Gr-1^lo^, MerTK^+^, CD206^+^, CD163^+^, and CX3CR1^hi^. Both subsets are CSF1R^+^, SIRPα^+^, CD64^+^, MHC II^+^, CD14^+^, and CD11b^+^. **(C)** Developmental dynamics of SG Mφ subsets. Embryo-derived Mφs dominate early development. In adulthood, both subsets are present, with Mo-derived Mφs becoming the major homeostatic population. Growth factors influence composition and function developmentally. **(D)** Disturbances in Mφ subset balance occur during inflammation/diseases. Under homeostatic conditions, tissues are maintained predominantly by long-lived Mφs. In contrast, inflammatory or disease states drive recruitment of short-lived Mφs, increasing their relative abundance and thereby contributing to tissue dysfunction. **(E)** Microenvironmental cues shape Mφ development and tissue adaptation. SG microenvironmental factors like chemokines and adhesion molecules facilitate Mo-derived Mφ recruitment. Cytokines such as CSF1 and IL-34 are essential for repopulation, differentiation, and proliferation of both subsets, influencing adaptation and function. Abbreviations: BM, bone marrow; EMPs, erythromyeloid progenitors; HSC, hematopoietic stem cell; Mo, monocytes. Image created with BioRender.com.

SG Mφs exhibit dynamic dual origins: embryonically from yolk sac/fetal liver progenitors, and postnatally from bone marrow (BM)-derived monocytes (31). Under homeostatic conditions, the precise mechanisms governing Mφ population maintenance and the relative contributions from different cellular origins remain contested, with two competing perspectives predominating in the literature. **Figure 2** systematically delineates the ontogeny and dynamic remodeling of SG Mφs. It illustrates a central principle: the equilibrium between long-lived, self-sustaining Mφs of embryonic origin and their short-lived counterparts, continuously replenished from bone marrow monocytes, is critically determined by developmental stage, pathological insults, and microenvironmental signals. This framework is fundamental to understanding both SG homeostasis and the pathogenesis of its disorders.

Early studies suggest that, under homeostatic conditions, SG Mφs are autonomous and primarily maintained through local self-renewal rather than continuous replenishment from BM or peripheral blood monocytes. Supporting evidence includes: minimal donor-derived Mφs detected in SGs following short-term BM transplantation (21 days) (20); the presence of substantial CCR2-independent, tissue-resident Mφ populations in steady-state SGs, with negligible contributions from CCR2^+^ monocyte-derived Mφs, even after an acute inflammatory challenge (TNF stimulation for 5 hours), the amount of SG resident Mφs (Gr-1^low^ CSF1R^+^) is stable with the main recruited cells being classical monocytes and neutrophils (32); as evidenced by unaltered numbers of SG Mφs in *Flt3l^-/-^* and *Ccr2^-/-^* mice, demonstrating their population is maintained independently of Flt3L and CCR2 signaling. This distinguishes them from conventional dendritic cells and inflammatory monocytes that require continuous replenishment. Besides, their population size remains stable four weeks after murine *Cytomegalovirus* (CMV) infection (33). Furthermore, SG stromal cell-derived CSF1 plays a crucial role in the development and function of tissue-resident Mφs (16, 34). Collectively, these findings highlight the prominent role of tissue-resident Mφs and local microenvironmental factors in maintaining the SG Mφ population during homeostasis. These foundational findings, however, were largely based on indirect evidence and lacked the resolution of long-term, definitive fate-mapping.

Contrasting with the above perspective, subsequent investigators that employed developmental timeline analyses and lineage-tracing technologies have documented a developmental shift in SG Mφ origins. Lu et al. demonstrated that CD11c^-^ embryo-derived, self-renewing resident Mφs predominate during embryonic and neonatal stages but diminish proportionally with age. Conversely, CD11c^+^ Mφs, which are scarce during embryonic development, proliferate postnatally and emerge as the dominant population in adult SGs (constituting approximately 80% by 4 weeks of age in mice). These CD11c^+^ Mφs originate from BM, with their migration and accumulation from peripheral blood monocytes occurring in a CCR2-dependent manner, as verified through BM transplantation experiments (**Figures 2A, B**) (21). The long-term, multimodal and complementary lineage tracing of McKendrick et al. (e.g., *Cdh5-CreERT2* for embryo (yolk−sac or HSC) derived and *Ms4a3-Cre* for BM progenitors)further provided direct evidence for the continuous and progressive replenishment and replacement of the foundational embryonic-derived SG Mφ pool by BM-derived cells during postnatal development, and demonstrates that while short-term replenishment (within 7 days) after injury (such as irradiation) relies on the proliferation of resident Mφs, the long-term outcome (3–6 months) is an accelerated replacement of the foundational pool by BM-derived cells (13). Thus, while embryonic precursors substantially contribute to the early SG Mφ pool, BM-derived monocytes progressively replace them throughout postnatal development, a process that can be markedly accelerated by injury (**Figure 2C**).

Crucially, this homeostatic balance undergoes dramatic shifts during inflammatory or pathological conditions (13). During inflammation or viral infection, peripheral monocytes rapidly infiltrate the SGs, differentiate into Mφs, and orchestrate the immune microenvironment prior to lymphocyte recruitment (35). These infiltrating monocyte-derived Mφs serve essential functions in disease states by facilitating the subsequent influx, differentiation, and activation of T and B lymphocytes (**Figures 2D, E**).

Taken together, the origin of SG Mφs is not static but is dynamically sculpted by developmental stage, microenvironmental cues, and, as recently discovered, sex. This sexual dimorphism fundamentally reframes SG Mφ dynamics within a paradigm of balance between long-lived, self-sustaining resident populations and short-lived, monocyte-derived counterparts (3, 36). This equilibrium is not static; rather, it is exquisitely tuned by physiological cues such as sex and profoundly perturbed by pathological insults like tissue injury (13, 37). Deciphering the mechanisms that establish, maintain, and disrupt this balance is therefore central to understanding both SG homeostasis and pathology. The apparent contradictions in the literature largely reflect an evolution in experimental methodology, from phenotypic snapshots (Short-term BM transplantation, flow cytometry-based phenotypic analysis) to high-resolution, long-term lineage tracing. Furthermore, functional heterogeneity may be linked to distinct replacement kinetics, with perivascular Mφ subsets potentially exhibiting greater longevity than their epithelial-associated counterparts. Critically, a recent preprint has introduced a pivotal variable that may reconcile many of these discrepancies: sexual dimorphism. This work suggests that male SG Mφs are predominantly long-lived and self-renewing, whereas the female Mφ compartment relies on continuous replenishment by short-lived monocytes (37). This groundbreaking finding not only provides a compelling framework for unifying previous data but also mandates the consideration of sex as a fundamental biological variable in all future studies of SG immunobiology. Additionally, the developmental timepoint analyzed (embryonic, neonatal, adult) and the physiological context (steady state versus injury/inflammation), including the specific timepoint of analysis post-insult (i.e., short-term recovery vs. long-term remodeling), drastically alter ontological readouts.

Synthesizing the available evidence, we propose that SG Mφs follow a "developmental transition model": embryonic-derived self-renewing Mφs predominate during embryonic and early developmental stages; with advancing age, BM-derived monocytes progressively become the primary source; and during pathological states, monocyte recruitment increases dramatically. This dynamic equilibrium is tightly regulated by tissue microenvironmental factors, sex-specific signals, and developmental stage. Future investigations should integrate long-term lineage tracing approaches, single-cell multi-omics platforms, and spatial technologies to elucidate the precise molecular mechanisms governing the dynamic heterogeneity of SG Mφs and enhance translational relevance from murine models to human SG immunobiology.

### 2.2 Niche-driven heterogeneity

**Figure 3:**
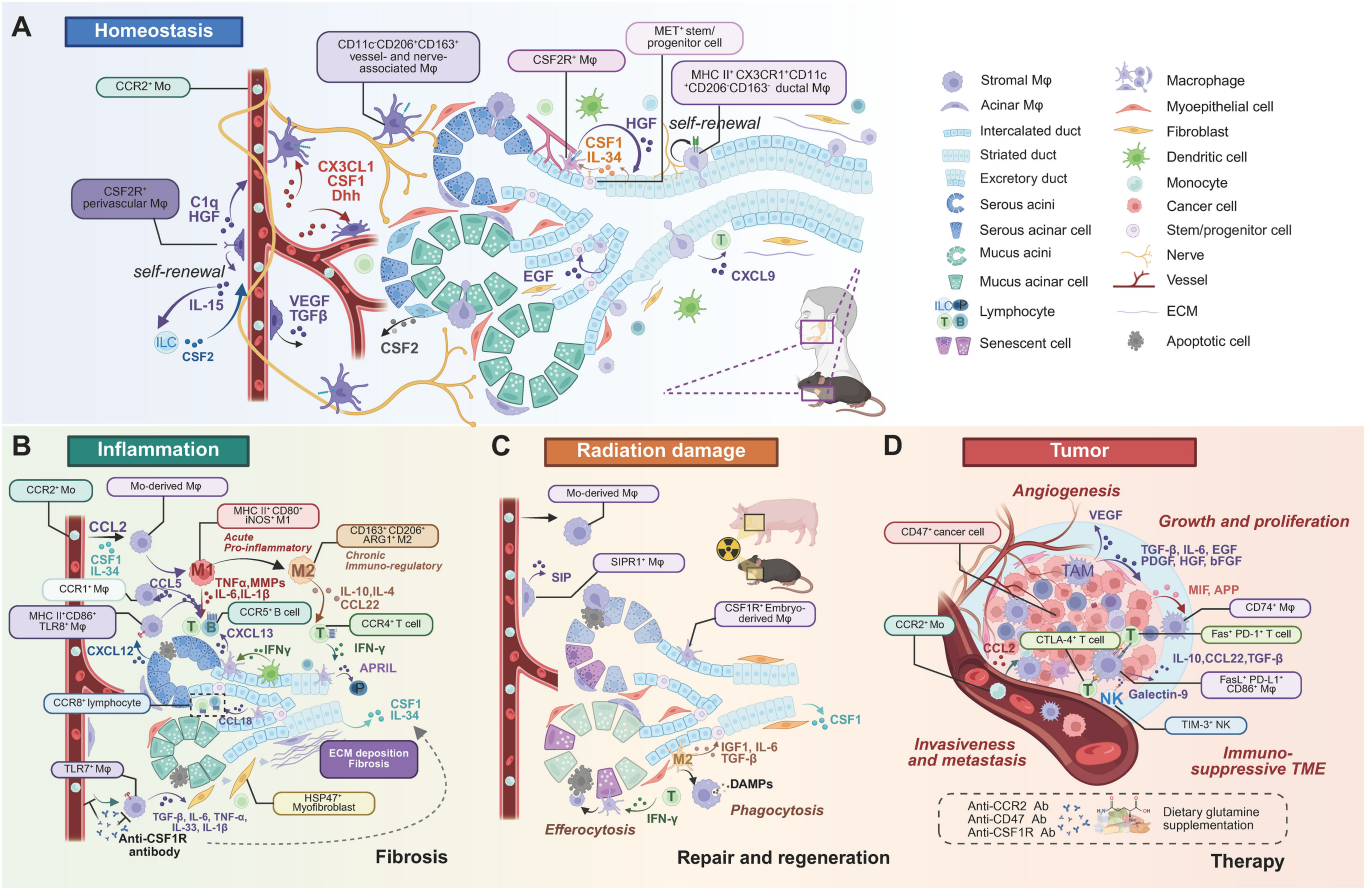
Heterogeneous subtypes, localization, and cell crosstalk of SG Mφs in homeostasis and diseases. **(A)** Homeostasis. Resident SG Mφs occupy distinct anatomical niches: self-renewing perivascular (CSF2R^+^), vessel/nerve-associated (CD11c^-^CD206^+^CD163^+^), and ductal (MHC II^+^CX3CR1^+^CD11c^+^CD206^-^CD163^-^). Mφs crosstalk with various cell types via cytokines, maintaining tissue homeostasis. **(B)** Inflammation and fibrosis. Inflammation involves Mo-derived Mφs infiltration. Acute inflammation features pro-inflammatory (M1-like) Mφs (MHC II^+^CD80^+^iNOS^+^) producing TNF-α, IL-1β, and MMPs, recruited and activated by acinar CXCL12. DCs and Mφs collaborate to recruit B and T cells (by IFN-γ, CXCL13, CCL22). Chronic inflammation involves immunoregulatory (M2-like) Mφs (CD163^+^CD206^+^ARG1^+^) promoting resolution and repair (by IL-4, IL-10, APRIL). Fibrosis involves CSF1-recruited Mo-derived Mφs and TLR7^+^ Mφs secreting pro-fibrotic factors (TGF-β, IL-6, TNF-α, IL-33, IL-1β) that stimulate myofibroblasts and ECM deposition. Anti-CSF1R therapy is a potential strategy. **(C)** Radiation damage, repair, and regeneration. Mo-derived Mφs and resident CSF1R^+^ Mφs respond to radiation. Perivascular SIPR1^+^ Mφs secrete SIP. Mφs clear damaged cells (efferocytosis, phagocytosis) and DAMPs, secreting pro-regenerative factors (IGF1, IL-6, TGF-β) that stimulate epithelial progenitors, promoting tissue repair. **(D)** Tumor progression and therapy. TAMs are major components of the immunosuppressive TME, typically M2-like, promoting progression through angiogenesis (by VEGF), growth (TGF-β, IL-6, EGF, PDGF, HGF, bFGF), and metastasis (CCL2 recruiting CCR2^+^ Mφs). TAMs interact with immune cells (NK, T cells) via various pathways (Galectin-9/TIM-3, CD86/CTLA-4, PD-L1/PD-1, FasL/Fas). Immune evasion via the CD47-SIRPα axis. Tumor cell-surface CD47 ligates the SIRPα receptor on Mφs, delivering an inhibitory "don't eat me" signal that prevents phagocytic clearance. Therapies target TAMs (anti-CCR2, anti-CD47, anti-CSF1R antibodies). Note: While depicted in a human context for relevance, most cellular crosstalk pathways have been primarily elucidated in murine models. Abbreviations: DAMPs, damage-associated molecular patterns; ECM, extracellular matrix; Mo, monocytes; TAM, tumor-associated Mφ; TME, tumor microenvironment.Image created with BioRender.com.

The functional diversity of SG Mφs is profoundly shaped by their microenvironmental niche, which dictates their phenotype and spatial organization far beyond the traditional M1/M2 classification (38). Advanced technologies have begun to unravel this remarkable heterogeneity in both health and disease (**Table 1**). **Figure 3** provides a panoramic view of the functional plasticity of salivary gland macrophages, illustrating how their subtypes, localization, and cellular interactions are dramatically remodeled across distinct physiological and pathological settings—from homeostasis and inflammation to tissue repair and malignancy. This highlights a central theme: the dialogue between macrophages and their niche is a critical determinant of glandular fate.

**Table 1:** SG Mφ subpopulation heterogeneity.

Subtype	Markers	Origin & Maintenance	Localization	Proportion (adults)	Functions	Study Model	Ref.
Embryonic-derived	CD11c^-^CD206^hi^MerTK^hi^ CX3CR1^lo^MHC II^hi(postnatal)/-(embryo)^	Embryonic-derived Self-renewal CSF1–dependent	Enriched in ductal regions	↓ with age (predominant in embryo/neonate)	Weak phagocytosis (CD11c^−^ > CD11c^+^) Immunomodulatory/Anti-inflammatory	Homeostasis	(21)
BM–derived	CD11c^+^CD206^lo^MerTK^lo^CX3CR1^hi^MHC II^hi^	BM–derived CCR2/CSF1–dependent	Interstitium & ducts	~80 %; ↑ postnatally	Weak phagocytosis/cross- presentation Promote gland development Reduced immune response		
Perivascular	CSF2R^+^CX3CR1^hi^TGFBR^hi^SiglecF^–^	Tissue-resident Self-renewal CSF2/Hedgehog-dependent	Around vessels & small ducts	Rare	Maintain tissue homeostasis Interact with ILCs Restore secretion function Phagocytosis; Anti-pathogen immunity	Homeostasis	(16)
Proliferative	*Cx3cr1, Stmn1*, *Birc5*	Tissue-resident Self-renewal		Low			
Nervous/epithelial-associated	MHC II^hi^CX3CR1^hi^	Tissue-resident Self-renewal Partial Mo input	Around nerves & large ducts		Regulate saliva secretion Phagocytosis; Anti-pathogen immunity		
Inflammatory infiltrated	CCR2^+^CX3CR1^lo^ADGRE1^lo^C1q^lo^LY6C^hi^	Mo-derived		~8%, while the others ~92%	Pro-inflammatory		
	CD11c^+^		Peri-epithelial/perivascular Near ~70% T cells	97.8% of CD11c^+^ cells are Mφ	Efferocytosis: Scaffold T cell patrolling; drive T cell clustering Traverse basal lamina to bridge epithelium	Homeostasis	(15)
Mo-derived (MDMs)	Gr-1^hi^	Mo-derived	Perivascular & parenchyma	↑ during inflammation	Drive immune responses Enhance vascular permeability/inflammatory infiltration	Homeostasis; inflammation	(32)
Tissue-resident	Gr-1^lo^	Tissue-resident		Predominant in homeostasis Stable	Maintain microvascular homeostasis/tissue stability		
M1-like	CD11b^lo^CD11c^lo^MHC II^lo^CD86^lo^CD206^lo^CD204^lo^CD36^lo^		Enriched at SjD lesions around ducts, acini and vessels	↑ early in SjD initiation		SjD	(39)
M2-like	CD11b^hi^CD11c^hi^MHC II^hi^CD86^hi^CD206^hi^CD204^hi^CD36^hi^	Tissue-resident		↑ with SjD progression	Enhance CCR4^+^ T cell migration/IFNγ production, breaking immune tolerance High phagocytosis/antigen presentation		
Immune regulation	*Apoe*			6.3% of SjD immune infiltrates	Immune regulation and lipid metabolism–related	SjD	(40)
Antigen-presentation	*Ifitm3, Fabp5, Ifi30*, *H2-Aa*				Enhanced antigen-presentation		
Activated	*Aif1, Trem2*				Activated state		
High pro-inflammatory	TLR8^+^	Mo-derived or possibly tissue-resident		↑ in SjD; Predominant TLR8+ immune cells	Release pro−inflammatory cytokines Activate T cells	SjD	(42)
	TLR8^-^						
	CCR1^hi^CCR5^hi^				recruit and activate CCL5^hi^T cells	SjD	(65)
	CX3CR1^+^ PDPN^+^	Tissue- resident	Parenchyma (early); Around TLS (late)	Homeostasis: ~48.7% of myeloid cells Acute inflammation: ↑ (peaks with TLS formation)	Pro-inflammatory Promote lymphangiogenesis and TLS formation/maintenance	SjD	(41)
M2-like	CD206^+^		Surrounding TLS (late)	↓, then recovers, peaks in late stage/TLS maturation	Anti-inflammatory, immune regulatory and tissue repair Maintain TLS stability		
Tissue-resident	CD11c^+^MHC II^hi^Ly6c^-^	Tissue-resident Flt3L-/CCR2-independent	Clustered Near infected acinar cells	Stable	Colocalize with T cells post- infection Drive T cell IFNγ response Lack cross-presentation	Homeostasis; infection	(33, 92)
Epithelial-associated	F4/80^+^		Limited migration Clustered, persistent contact with epithelial progenitors	High distribution density	Support tissue homeostasis (phagocytosis of apoptotic cells), promoting epithelial repair	Homeostasis; radiation injury	(45)
Epithelial-associated	CD11c^+^CD206^-^CD163^-^	BM-derived; Rapid turnover CSF1/CSF1R-dependent	Epithelium & T cell zones.	Predominate in adults	Eliminate DNA-damaged cells Facilitate epithelial regeneration	Homeostasis; radiation injury	(13)
Neuro/Vascular-associated (TLF Mφ)	CD11c^-^FRβ^+^ CD206^+^CD163^+^Tim4^+^LYVE1^+^	Embryonic-derived; long-lasting Self-renewal	Perivascular & perineural	Neonatal−predominant	Regulate local immunity/tissue homeostasis		
Proliferative	*Ki67, Tubb5, Top2a*	Self-renewal		Low Rapid ↓, soon proliferation restores			
Tissue-remodeling	*Vim*, *Mmp12*, *Fn1*			Normal SG residents ↑ in tumors (recruitment/proliferation)	Tissue remodeling	Squamous cell carcinoma	(44)
Proliferative	*Hmgb2, Mki67*	Self-renewal			Proliferating		
Interferon-mediated	*Isg15, Rsad2, Irf7*				Interferon response		
Inflammatory	*Irg1*, *S100a9*, *S100a8*				Inflammation		
Inflammatory IL1B^+^ (M1-like)	*IL-1β, BCL2A1, PSMA2, TNFRSF1B, FCGR2A*			Present in normal tissue	Drive inflammation; activate neutrophils Antigen presentation and intercellular interactions	Normal; Adenoid cystic carcinoma	(43)
EMT-associated (M2-like)	*CCL3, CCL4, CD163, MSR1, CTSC, CTSD, FGL2, HEXB, MAF, MS4A6A, SLC4A7*			↑ in tumors	Promote tumor invasiveness/metastasis		

MDM, monocyte-derived Mφ; Mo, monocytes; TLS, tertiary lymphoid structures.↑, increase; ↓, decrease.

Under homeostasis, distinct SG Mφ subpopulations have been identified based on unique markers and niche localization (**Figure 3A**). Early studies identified CD45^+^MHC II^+^ cells expressing Mφ markers (33), later confirmed to be predominantly CD64^+^ Mφs, many of which also express CD11c (13, 15). Key subsets include CCR2^-^dependent, BM-derived CD11c^+^ Mφs that are prevalent in adults and closely interact with epithelial cells, and their embryonically-derived CD11c^-^ counterparts that dominate in neonates (21). Further distinction is made by Gr-1 expression, which separates infiltrating monocyte-derived Mφs (Gr-1^hi^) from tissue-resident Mφs (Gr-1^lo^) (32) (**Figure 2B**).

Single-cell analyses have provided even greater resolution, revealing multiple CX3CR1^+^ Mφ populations with distinct transcriptomes and locations. These include CD11c^+^CD206^-^ Mφs associated with the epithelium and potentially inflammatory roles, and CD11c^-^CD206^+^ Mφs associated with blood vessels and nerves that are implicated in tissue repair (13). Additional complexity is evident with the identification of self-renewing, nerve- and large duct-associated CX3CR1^hi^ MHC II^hi^Mφs and vessel-associated CX3CR1^hi^CSF2R^+^ Mφs, each with specific homeostatic functions, alongside a minor population of pro-inflammatory CCR2^+^ monocyte-derived Mφs (16) (**Figure 3A**).

This heterogeneity is further amplified in disease states (**Table 1**). In SjD, Mφs are broadly divided into CD11b^hi^ (M2-like) and CD11b^lo^subsets, with the former linked to T-cell responses via CCL22 (39). ScRNA-seq has identified multiple Mφ subclasses in SjD models with roles in immunity, metabolism, and inflammation, as well as dynamic changes in resident Mφ populations (CX3CR1^+^PDPN^+^ and CD206^+^PDPN^+^) during disease progression (40, 41). In SjD patients, TLR8^+^ Mφs are implicated in driving pathogenesis (42) (**Figure 3B**). In SG adenoid cystic carcinoma (ACC), Mφ infiltration is reduced, but distinct clusters, including inflammatory M1-like and pro-invasive epithelial-mesenchymal transition (EMT) -associated Mφs, have been identified (43). Murine SG tumors likewise exhibit multiple tumor-associated Mφ (TAM) subpopulations with diverse functions (44).

In summary, SG Mφs exhibit dual embryonic and bone marrow origins with dynamic composition shifts during development and disease states. Their heterogeneity extends beyond the classical M1/M2 paradigm, with distinct niche-specialized subpopulations identified through advanced single-cell technologies. Recent evidence reveals surprising sexual dimorphism in Mφ ontogeny and maintenance. Human validation of Mφ subsets identified in murine models remains limited, hindering translational applications. Specific molecular mechanisms governing subtype-specific functional specialization are poorly understood. Tools for targeted fate-mapping and manipulation of individual SG Mφ subsets are lacking, particularly for studying dynamic transitions during pathological conditions. The functional significance of sex-specific differences in Mφ ontogeny warrants further investigation, especially in sex-biased disorders like SjD.

## 3 Mφ-niche crosstalk in SGs

Within the SG niche, the precise spatial arrangement of Mφs, stromal cells, and other immune cells facilitates critical reciprocal interactions. This dynamic, bidirectional signaling network, mediated by both direct cell-cell contact and paracrine factors, is essential for coordinating SG Mφ surveillance, rapid inflammatory responses, and ultimately, maintaining glandular homeostasis. These interactions, whether through direct contact via surface molecules or broader paracrine communication, profoundly shape the functional organization of the SG microenvironment.

### 3.1 Mφ–epithelial crosstalk

Mφs engage in intricate crosstalk with the acinar and ductal epithelial cells that govern saliva production. Imaging studies reveal Mφs in close association with epithelial structures, extending protrusions that interact with epithelial cells (EpCAM^+^) and phagocytosing apoptotic cells after injury (15, 45). This communication is critical for tissue maintenance and regeneration. Resident Mφs secrete factors like hepatocyte growth factor (HGF) to promote the regeneration and DNA repair of MET^+^ epithelial progenitors, while acinar cells release CSF2 to induce a regenerative Mφ phenotype (3, 16, 46, 47). This reciprocal support is further evidenced by Mφ-derived epidermal growth factor (EGF) amplifying acinar progenitors and epithelial-derived CSF1 and IL-34 sustaining the CSF1R^+^ Mφ population (16, 31, 48) (**Figure 3A**).

During inflammation, this crosstalk intensifies. Activated epithelial cells can stimulate Mφs via TLR signaling to produce TNF-α, and the release of CXCL12 by inflamed epithelia enhances Mφ recruitment for tissue repair (13, 49). In SjD, activated epithelial cells recruit Mφs and other innate immune cells, which in turn release inflammatory mediators (e.g., IL-1, TNF-α, MMPs) that contribute to tissue damage (50).

### 3.2 Mφ-stromal crosstalk

Mφs also maintain extensive communication with stromal components, including nerves, blood vessels, and fibroblasts, to support tissue structure and function.

#### 3.2.1 Mφ-nerve interactions

The neuroimmune axis is critical for SG homeostasis and repair. A key example is the parasympathetic-Mφ-epithelial cell axis, where parasympathetic stimulation induces Mφs to secrete IL-6, which promotes ductal cell proliferation and gland regeneration via STAT3 phosphorylation (51). Spatially, MHC II^hi^ SG Mφs are found in close proximity to nerve fibers, resembling nerve-associated Mφs in other organs and suggesting specialized functional interactions (**Figure 3A**) (16). Disruption of this axis, such as through impaired neural innervation in SjD or potential sex-specific differences in Mφ-nerve interactions, may contribute to glandular pathology (37, 52, 53).

#### 3.2.2 Mφ-vessel interactions

SG function depends on a dense microvascular network, and perivascular Mφs are crucial sentinels that regulate vascular integrity. Subsets like CSF2R^+^ Mφs and CD11c^-^CD206^+^CD163^+^ Mφs are closely associated with the vasculature, interacting with endothelial cells via factors such as C1q, HGF, and CSF1 (**Figure 3A**) (13, 16, 54). Furthermore, Hedgehog pathway activation in these Mφs can stimulate VEGF secretion to promote angiogenesis (46). During inflammation, Mφ plasticity determines vascular outcomes: M1 Mφs increase vascular permeability, whereas M2 Mφs support vascular repair and resolve inflammation (55).

#### 3.2.3 Mφ-fibroblast interactions

In fibrotic SG diseases, the interplay between Mφs and fibroblasts is a key driver of pathology. In IgG4-related disease (IgG4-RD), CD163^+^ M2 Mφs promote fibroblast activation and fibrosis by releasing profibrotic cytokines (e.g., IL-33, TGF-β) through the TLR7/IRAK4/NF-κB pathway (56). Similarly, in chronic graft-versus-host disease (cGVHD), Mφs secrete TGF-β and other pro-inflammatory cytokines that induce myofibroblast differentiation, creating a feedback loop that exacerbates fibrosis (**Figure 3B**) (57, 58). This pro-fibrotic crosstalk is also central to the tumor microenvironment, where cancer-associated fibroblasts (CAFs) and tumor-associated Mφs (TAMs) collaborate to drive tumor progression (59).

### 3.3 Mφ-immune cell crosstalk

As a prominent innate immune population, Mφs orchestrate the local immune landscape through extensive interactions with other immune cells (16, 22, 23, 60).

Under homeostatic conditions, SG Mφs guide the surveillance patterns of tissue-resident memory T cells (TRM), which in turn stimulate Mφ phagocytic activity via IFN-γ (55). This is facilitated by Mφ-secreted CXCL9, which attracts CD8^+^ TRMs into ductal "patrolling clusters". Mφ depletion impairs TRM motility, underscoring this cooperative surveillance relationship (15). Furthermore, Mφs establish a critical homeostatic loop with innate lymphoid cells (ILCs), where Mφ-derived IL-15 sustains ILCs, and ILC-derived CSF2 maintains the CSF2R^+^ Mφ population (**Figure 3A**) (16).

In disease, these interactions become amplified and pathogenic. In ACC, tumor progression may be driven by heightened MIF-CD74 and APP-CD74 signaling between epithelial cells and Mφs (43). In autoimmune diseases like SjD, Mφs are central to the inflammatory infiltrate (61). They function as antigen-presenting cells and, upon TLR8 activation, express co-stimulatory molecules like CD86 to activate T cells (42). Mφs collaborate with plasmacytoid dendritic cells (pDCs); pDC-derived type I IFN stimulates Mφs to produce CXCL13, which recruits B cells (62, 63). Additionally, Mφ-secreted CCL22 promotes T-cell migration and IFN-γ production, contributing to the breakdown of immune tolerance (**Figure 3B**) (39, 64). Targeting these interactions, such as the CCR1/CCL5 chemokine axis that mediates Mφ, T cell, and B cell crosstalk, has shown therapeutic potential in preclinical SjD models (65). In IgG4-RD, distinct chemokine axes, including CCL18–CCR8, are upregulated, and M2 Mφ-derived APRIL contributes to plasma cell accumulation (66, 67).

In conclusion, SG Mφs are not just immune cells but key "communication hubs" within the tissue niche, engaged in bidirectional communication with epithelial, stromal, and immune cells through both contact-dependent mechanisms and paracrine signaling. These interactions are critical for tissue homeostasis, with specialized Mφ subsets serving as regulatory hubs at epithelial, vascular, and neural interfaces. During disease states, these communication networks become dysregulated, driving pathological processes including inflammation, fibrosis, and tumor progression. However, our current understanding is largely based on static depictions of individual signaling axes, limiting our system-level perception. The temporal dynamics and spatial organization of Mφ-niche interactions remain poorly characterized, particularly in real-time *in vivo* settings. The molecular mediators facilitating communication between Mφs and specific SG cell types require further delineation. Comparative analyses between human and murine SG Mφ communication networks are limited, constraining translational insights. How age and sex influence these niche interactions and their subsequent impact on disease susceptibility represents a critical area for future investigation.

## 4 Physiological roles of SG Mφs: guardians of tissue integrity and mediators of immune surveillance

In their homeostatic state, SG Mφs function as critical sensors and effectors, supporting tissue integrity and orchestrating immune surveillance through finely tuned interactions with their microenvironment. Constituting approximately 90% of the antigen-presenting cell (APC) population in murine submandibular glands, their dominant presence underscores a conserved role as guardians of glandular function, analogous to their counterparts in other exocrine glands like the mammary gland (21, 68). Functionally, glandular homeostasis is not executed by a monolithic Mφ population but emerges from a synergistic interplay between long-lived and short-lived subsets. Long-lived resident Mφs act as steadfast "guardians," orchestrating tissue architecture, immune surveillance, and local tolerance through their intimate and stable crosstalk with the epithelial and stromal niche (16). Concurrently, short-lived, monocyte-derived Mφs function as dynamic "sentinels," providing the gland with the capacity for rapid responses to acute challenges (21). This finely tuned balance between guardianship and vigilance ensures the remarkable resilience of SGs within the challenging oral milieu.

### 4.1 Driving glandular development and governing secretory epithelial homeostasis

Tissue-resident Mφs are key regulators of organogenesis in branching organs, and evidence suggests they contribute to SG development. In the embryonic mouse SG, CD11c^-^ Mφs are present by E13.5 and localize to ductal regions, where they may guide branching morphogenesis, a role established for Mφs in the lungs and mammary glands (21). CSF1-dependent Mφs are integral to this process in other branching organs (69–71), and in SGs, CSF2R^+^ resident Mφs are a key source of HGF, which regulates epithelial progenitor homeostasis (16). Furthermore, signaling molecules like CSF1 and BMP4 have been shown to modulate branching, with BMP4 potentially recruiting Mφs to morphogenic sites (**Figure 2C**) (72, 73). However, the precise role of Mφs remains complex, as postnatal systemic Mφ depletion did not significantly alter SG morphology, suggesting the existence of compensatory mechanisms (21).

### 4.2 Regulated phagocytic and efferocytic activity

While essential phagocytes, SG Mφs exhibit tightly regulated activity in the steady state. Compared to peritoneal Mφs, they show surprisingly low baseline phagocytic gene expression, suggesting their primary homeostatic function is not phagocytosis but rather the modulation of glandular functions (21). This basal state is poised for activation, as their phagocytic capacity increases during inflammation. Furthermore, SG Mφs are equipped for efferocytosis—the clearance of apoptotic cells to maintain tissue equilibrium and immune tolerance—through the expression of TAM receptors (*Tyro3, Axl, Mertk)* (**Figure 3C**) (74–76). Their potential involvement in preventing crystal formation, similar to the role of renal Mφs in preventing kidney stones, remains an important but unexplored area in sialolithiasis research (77).

### 4.3 Facilitating immune surveillance

As a mucosal barrier, the SGs are a frequent target for pathogens, making immune surveillance a critical physiological function (22, 78–82). SG Mφs are central to this defense network. They directly influence the motility and epithelial-patrolling behavior of tissue-resident memory CD8^+^ T cells (TRMs), which are essential for long-term protection against local infections (15). This Mφ-TRM cooperation establishes a highly effective surveillance system within the gland. Additionally, Mφs support other innate defenders; for instance, the local ILC population, which is dominated by defensive ILC1 and NK cells, depends on a steady supply of IL-15 provided by CSF2R^+^ Mφs (**Figure 3A**) (16, 83).

### 4.4 Enhancing microvascular homeostasis

The dense microvascular network of the SGs is vital for its secretory function, and Mφs are key regulators of its homeostasis. Specific Mφ subpopulations, such as perivascular CSF2R^+^ Mφs and CD11c^-^CD206^+^CD163^+^ Mφs, are strategically localized in close contact with blood vessels (**Figure 3A**) (13, 16). From this perivascular niche, they interact with endothelial cells via factors like C1q to sense microenvironmental changes and modulate vascular function, ensuring tissue stability and a rapid response to injury (16, 32).

In essence, beyond traditional immune functions, SG Mφs serve as essential regulators of secretory epithelial homeostasis and microvascular integrity. They orchestrate immune surveillance through cooperation with tissue-resident lymphocytes, while exhibiting tightly regulated phagocytic and efferocytic activities that balance defense with tissue protection. The functional equilibrium between long-lived "guardian" and short-lived "sentinel" Mφ populations is fundamental to SG resilience. The precise contributions of Mφs to SG morphogenesis remain incompletely understood, with contradictory findings regarding their necessity for normal development. Mechanisms governing the restrained phagocytic capacity of resident Mφs under homeostatic conditions are undefined, as are the triggers that enhance this activity during inflammation. The potential role of SG Mφs in preventing sialolithiasis through crystal clearance, analogous to their function of preventing kidney stones (77), represents an unexplored research direction. Cross-species validation of these physiological functions in human SGs is critically needed. Crucially, whether these precise physiological functions are conserved in humans remains largely speculative. The absence of human single-cell data from key developmental and pathological states, such as post-radiation injury, prevents the direct validation of these murine-defined roles and represents a critical bottleneck for clinical translation.

## 5 SG Mφs in diseases

Beyond their homeostatic functions, SG Mφs are pivotal players in the pathogenesis and resolution of glandular diseases. They adopt diverse, context-dependent roles in infection, inflammation, tissue repair, and cancer, highlighting their potential as therapeutic targets. Emerging evidence posits that a central pathogenic hub across diverse SG disorders is the disruption of the delicate equilibrium between long-lived, homeostatic Mφs and their short-lived, inflammatory counterparts. Insults ranging from external radiation damage to intrinsic processes like aging and autoimmunity appear to converge on a common pathology: the depletion and dysfunction of protective resident Mφs, coupled with an overwhelming influx and activation of monocyte-derived cells (13, 84). This imbalance is not a mere epiphenomenon but a critical driver that fuels a vicious cycle of inflammation, tissue injury, and irreversible functional decline.

### 5.1 Anti-infective immunity

As a gateway to the oral cavity, SGs are a critical barrier against pathogens like *Staphylococcus aureus* and various viruses, including CMV, *Lymphocytic choriomeningitis virus* (LCMV), and SARS-CoV-2, which can establish persistent infections and contribute to systemic disease (22, 25, 78–82, 85, 86). Mφs are central to this anti-infective response, yet their role can be a double-edged sword. In CMV infection, for instance, Mφs are exploited as vehicles for viral dissemination and replication (87–91). Immune control of CMV in the SGs is distinct from other organs, as local APCs (90% Mφs) lack cross-presentation capacity, and infected acinar cells downregulate MHC I, rendering CD8^+^ T cells ineffective (26, 33, 92). Consequently, viral control in the SGs relies heavily on CD4^+^ T cells and IFN-γ (26, 33). Despite this, Mφs contribute to antiviral defense; their depletion increases viral loads, and they are essential for facilitating the immune surveillance of remaining CD8^+^ T cells (15, 93).

### 5.2 Orchestration of inflammation and autoimmunity

SG Mφs are critical regulators of inflammation, exhibiting remarkable plasticity that allows them to drive both pro-inflammatory and reparative responses. In autoimmune diseases, this balance is disrupted, and Mφs become key mediators of pathology.

#### 5.2.1 Mφ plasticity and immune cell recruitment in autoimmunity

In conditions like SjD and cGVHD, the inflammatory microenvironment drives Mφs toward a pro-inflammatory M1-like phenotype. These cells are prominent in inflammatory lesions and secrete cytokines like TNF-α, IL-6, and CXCL13, which fuel tissue damage and correlate with disease severity (53, 61, 62, 94). In SjD, Mφ infiltration often precedes lymphocyte infiltration, suggesting they play a role in initiating the autoimmune cascade (94). This pathogenic role is underscored by findings that Mφ depletion improves glandular function in disease models.

The functional polarization of Mφs is highly dynamic and represents a key therapeutic target. While M1 Mφs dominate early SjD lesions, M2-like Mφs become more prominent as the disease progresses, initially contributing to resolution but ultimately mediating the irreversible fibrosis seen in advanced stages (61, 62, 95, 96). In IgG4-related disease, TLR7^+^ CD163^+^ M2 Mφs directly drive fibrosis by secreting TGF-β and other profibrotic factors via the TLR7/IRAK4/NF-κB pathway (**Figure 3B**) (56). This M1/M2 balance can be modulated by various interventions, including therapeutics like metformin and aspirin-triggered resolvin D1, which promote a shift toward the anti-inflammatory M2 phenotype (97, 98).

Beyond direct cytokine effects, Mφs orchestrate the adaptive immune response by recruiting and activating other immune cells. In SjD, TLR8 expression is upregulated on Mφs, which present antigens and express co-stimulatory molecules like CD86 to activate T cells (42). Mφs collaborate with pDCs, responding to type I IFN by producing CXCL13, which recruits CXCR5^+^ B cells into the gland (62, 63). Furthermore, Mφ-secreted CCL22 enhances T cell migration and IFN-γ production by upregulating CCR4 on T cells, a key step in breaking local immune tolerance (**Figure 3B**) (39). Targeting these chemokine axes, such as the CCR1/CCL5 loop mediating Mφ-T/B cell crosstalk, has shown therapeutic promise in preclinical models (65).

#### 5.2.2 Crosstalk with the oral microbiome

The oral microbiome can directly influence the SG immune microenvironment. For example, ligation-induced periodontitis in mice alters the oral microbiota, which correlates with an increase in the number and activation of SG Mφs (99).

#### 5.2.3 Regulation by cellular processes: autophagy, efferocytosis, and cuproptosis

Fundamental cellular processes in Mφs are linked to SG health and disease. In age-related SG dysfunction, impaired Mφ autophagy, regulated by the SIRT6-PI3K/AKT/mTOR pathway, leads to M1 polarization and hyposalivation (84). Similarly, defective efferocytosis, potentially due to dysfunctional TAM receptors (MerTK), is implicated in the accumulation of apoptotic cells and elevated type I IFN signaling in SjD (75, 76). Furthermore, cuproptosis, a form of copper-dependent cell death, has emerged as a potential mechanism in SjD, with cuproptosis-related genes being highly expressed in activated Mφs and other immune cells in affected tissues (100).

### 5.3 Roles in tissue repair and regeneration

The immune system plays a crucial role in tissue repair and should be integrated into the design of regenerative medicine treatments. Mφs are essential for successful tissue repair, significantly modifying their phenotype in response to microenvironmental cues and executing different functions throughout all stages of the repair process. In particular, based on their response to various stimuli, they can release growth factors, support angiogenesis, and promote extracellular matrix remodeling. Specifically, Mφs are indispensable for successful tissue repair, dynamically altering their phenotype to orchestrate all stages of the healing process (31).

#### 5.3.1 Post-radiation recovery

Radiotherapy causes severe damage to SGs, and Mφs are critical for subsequent recovery. Following irradiation, Mφs rapidly clear damaged and senescent epithelial cells (45, 101). This process is dependent on CSF1R^+^ Mφs, whose depletion severely impairs tissue repair (13). Resident Mφs are particularly important, and therapeutic strategies that preserve or activate them, such as transient Hedgehog pathway activation or local administration of sphingosine-1-phosphate (S1P), can restore Mφ function, promote angiogenesis, and rescue salivary function (16, 46, 47, 54) (**Figure 3C**). Furthermore, transplantation of *ex vivo*-conditioned M2-like Mφs, termed effective-mononuclear cells (E-MNCs), has shown considerable clinical potential by clearing damage-associated molecular patterns (DAMPs) and secreting regenerative factors such as IGF1 (102–104). A recent clinical trial in Japan (jRCTb070190057) has corroborated the therapeutic potential of E-MNCs in patients with radiation-induced xerostomia, showing significant functional improvement and reduced fibrotic burden (**Table 2**).

**Table 2:** Clinical trials of Mφ-based strategies in SG diseases.

Trial ID	Therapeutic Agents	Targets	Mechanism	Diseases/Conditions	Ref.
NCT02216409	Hu5F9-G4	CD47	Enhances Mφ phagocytosis via CD47-SIRPα blockade	Advanced solid tumors (including SG cancer or head and neck cancer)	(136)
NCT02734433	Pexidartinib	CSF1R	Inhibits TAMs survival	Advanced solid tumors (Pleomorphic adenocarcinoma, Adenoid cystic carcinoma)	(137)
NCT02526017	Cabiralizumab+Cabozantinib	CSF1R MET, VEGFR, AXL	Inhibits TAM survival Inhibits angiogenesis, proliferation, and metastasis.	Advanced solid tumors (Head and neck cancer)	
NCT06048367	CNSI-Fe(II)	Mφ polarization GPX4-regulated ferroptosis	Reprograms TAMs to an anti-tumor M1 phenotype through iron overload Induces tumor cell ferroptosis via iron-mediated ROS.	Advanced solid tumors (including head and neck cancer)	
jRCTb070190057	E-MNCs:	Inflammation Fibrosis	Delivering immunomodulatory M2 Mφs to suppress T-cell mediated inflammation secrete regenerative factors (e.g., IGF1) promote vasculogenesis reduce fibrosis	Radiation-induced xerostomia	(102–104)

AXL, AXL receptor tyrosine kinase; CNSI-Fe(II), carbon nanoparticle-loaded Iron; E-MNCs, effective-mononuclear cells; MET, mesenchymal-epithelial transition factor.

Mφ-targeted therapies for SG pathologies remain at an early and exploratory stage, with a limited dedicated clinical evidence base. All listed trials are reported as completed, and mostly involve general solid tumors rather than those specifically designed for SG cancers. Notably, a comprehensive search did not identify relevant trials for SjD or cGVHD. Future trials incorporating SG-specific endpoints and patient cohorts are warranted to establish efficacy in these unique disease contexts. Data are current as of September 2025.

#### 5.3.2 Post-inflammatory repair

In non-radiation injury models, such as obstructive injury, Mφs also mediate repair and fibrosis. M2 Mφs become enriched and promote tissue remodeling through the secretion of factors like TGF-β (12). In some contexts, foam Mφs are implicated in clearing necrotic debris, highlighting their diverse roles in both pathological and reparative processes (105–109).

### 5.4 Driving tumor progression

TAMs are critical components of the SG tumor microenvironment, often constituting a significant portion of stromal cells (30-50%). Their abundance inversely correlates with therapeutic response and survival. Predominantly displaying a pro-tumoral CD163^+^ M2 phenotype, TAMs in SG tumors uniquely promote tumor progression through proliferation, metastasis, angiogenesis, immune evasion, and recurrence, notably lacking typical phagocytic activity towards tumor cells (110, 111) (**Figure 3D**). ScRNA-seq reveals TAMs as a heterogeneous population with distinct subpopulations exhibiting diverse functional states beyond a simple pro- or anti-tumor dichotomy (44). Targeting TAMs holds promise as a complementary therapeutic strategy in various cancers, and its potential is being explored in SG tumors.

#### 5.4.1 Promoting an immunosuppressive microenvironment

TAMs, which are predominantly polarized to an M2-like phenotype, are instrumental in creating an immunosuppressive tumor microenvironment (TME) that facilitates tumor immune evasion (112). In salivary duct carcinoma (SDC), M2-like Mφs suppress T and NK cell activity via immune checkpoints like TIM-3/galectin-9 (113). Similarly, M2 Mφs are associated with immunosuppression in mucoepidermoid carcinoma (MEC) and ACC (114, 115).

A pivotal mechanism of immune evasion is orchestrated by the CD47-SIRPα axis, whereby tumor cells upregulate the "don't eat me" signal CD47 to engage the inhibitory receptor SIRPα on tumor-associated Mφs (TAMs) and suppress phagocytosis. However, the landscape within SG carcinomas (SGCs) presents a more complex scenario. Analysis across major SGC subtypes—including salivary duct, adenoid cystic, and mucoepidermoid carcinomas—reveals that CD47 expression is prominent not only on malignant cells but also on tumor-infiltrating immune cells (TIICs) (116). In mucoepidermoid carcinoma, for instance, CD47 expression on TIICs was found to be significantly higher than on tumor cells. This critical finding underscores that while therapeutic blockade of the CD47-SIRPα checkpoint is a promising strategy to unleash TAM phagocytic activity, its clinical implementation in SGCs warrants careful consideration of its potential impact on the resident immune infiltrate.

Furthermore, autophagy within the TME can dampen anti-tumor immunity by promoting the infiltration of Mφs and regulatory T cells (Tregs), an effect that can be partially countered with dietary glutamine (117) (**Figure 3D**).

#### 5.4.2 Facilitating tumor proliferation, invasion, and metastasis

Beyond immunosuppression, TAMs directly promote tumor growth, angiogenesis, and metastasis. In ACC, a key signaling axis involves tumor-derived CCL2 recruiting CCR2^+^ TAMs, which in turn secrete GDNF to drive tumor cell proliferation via the RET pathway (118) (**Figure 3D**). In MEC, TAM abundance correlates with tumor grade and size, and co-culture with Mφs enhances cancer cell invasion (119). TAMs achieve this by secreting a host of growth factors (e.g., EGF, PDGF, TGF-β) and collaborating with cancer-associated fibroblasts (CAFs) to promote neovascularization and metastasis (110, 120).

Ultimately, SG Mφs are master regulators of disease outcomes, with their function context-dependent. An emerging paradigm is that pathology stems from an imbalance between resident and monocyte-derived populations. Key obstacles are the lack of longitudinal data tracing subset dynamics during disease and the nascent state of Mφ-targeted therapies for SG-specific conditions. The path forward requires detailed time-resolved mapping of Mφ subsets in disease and the initiation of dedicated clinical trials that move beyond broad solid tumor cohorts to explicitly target SG disorders like SjD and radiation-induced damage.

### 5.5 Sex and age sculpt pathogenic SG Mφ responses

SjD presents a stark demographic bias, with a mean age of onset 51.7 years and a female predominance 93.4% (4). This clinical reality firmly establishes sex and age as critical variables in salivary gland pathology, where SG Mφs are emerging as key cellular mediators. A compelling "double-hit" hypothesis, integrating recent discoveries, may explain this demographic bias.

The "first hit" stems from a fundamental sexual dimorphism in Mφ ontogeny. Female mice depend on continuous replenishment by pro-inflammatory, short-lived "sentinel" monocytes, which establishes a baseline of heightened immune reactivity. This contrasts sharply with males, whose glands are populated by long-lived, self-renewing "guardian" Mφs that maintain homeostasis (37). The "second hit" arises with aging. In aged glands of both humans and mice, impaired macrophage autophagy—regulated by the SIRT6-PI3K/AKT/mTOR axis—skews Mφs toward a pro-inflammatory M1 phenotype that directly causes hyposalivation (84).

Together, these findings suggest that in older women, the female-intrinsic "sentinel" Mφ landscape is synergistically amplified by age-driven M1 polarization. This convergence likely creates a microenvironment ripe for breaking immune tolerance and initiating autoimmunity. This framework, however, is built largely on murine studies. A critical future direction is to validate these mechanisms in human SjD cohorts, using single-cell and spatial omics to dissect precisely how sex and age intersect to shape the pathogenic evolution of the human SG Mφ landscape.

Ultimately, SG Mφs are master regulators of disease outcomes, with their function being context-dependent. An emerging paradigm is that pathology stems from an imbalance between resident and monocyte-derived populations. However, key obstacles remain, including the lack of longitudinal data tracing subset dynamics during disease and the nascent state of Mφ-targeted therapies for SG-specific conditions. The path forward requires detailed time-resolved mapping of Mφ subsets and a dedicated effort to unravel the molecular mechanisms by which fundamental biological variables like sex and age impact macrophage function. This foundational knowledge is critical for the initiation of dedicated clinical trials that move beyond broad solid tumor cohorts to explicitly target SG disorders like SjD and radiation-induced damage.

## 6 Mφ-based therapies and biomarkers in SG diseases

**Figure 4:**
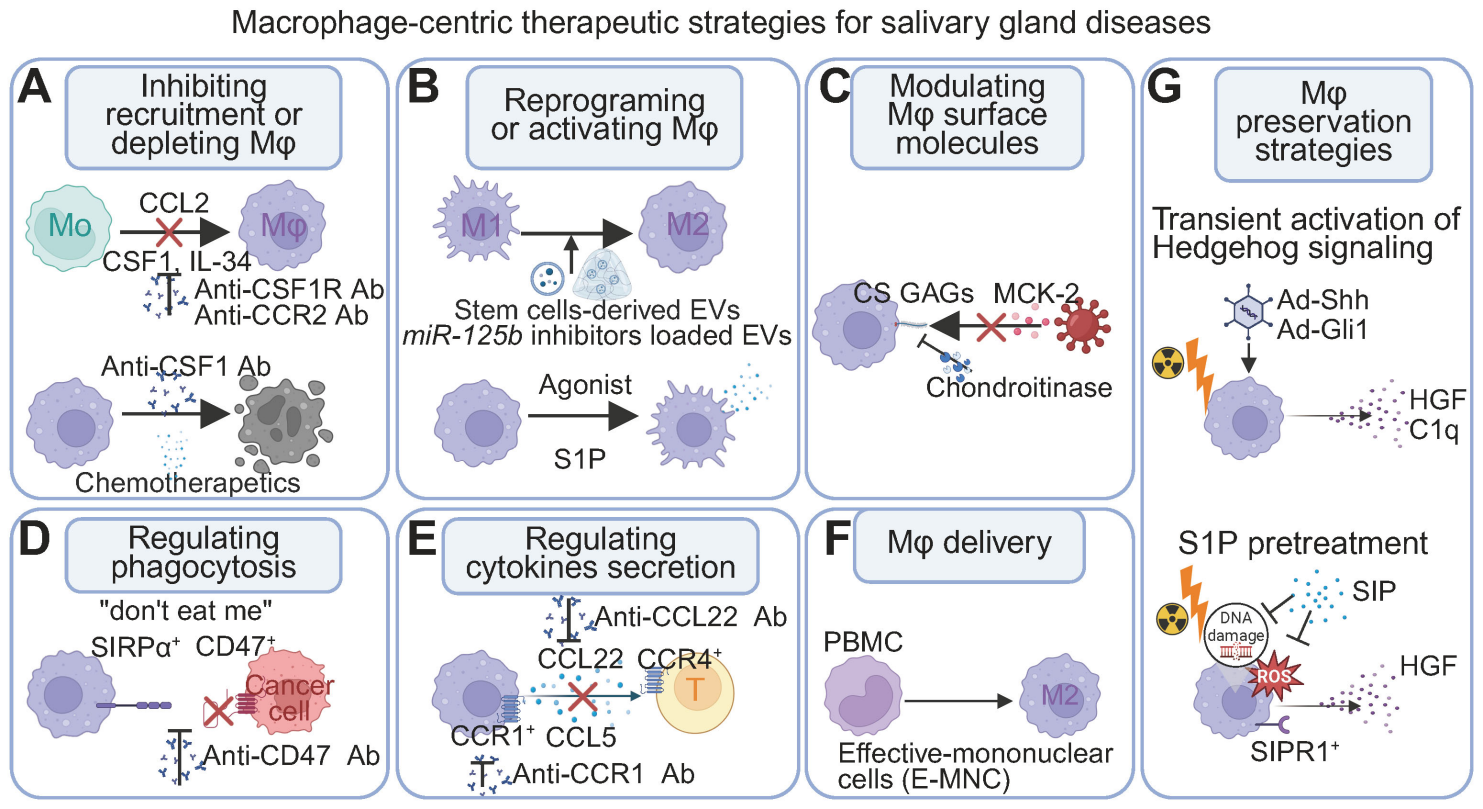
Mφ-centric therapeutic strategies for SG diseases. **(A)** Modulating abundance: Inhibiting monocyte recruitment (e.g., blocking CCL2-CCR2), interfering with the differentiation and survival of macrophages (e.g., blocking the CSF1/IL-34 - CSF1R axis), or depleting existing populations (e.g., chemotherapeutic agents). **(B)** Reprogramming function: Shifting polarization or activating therapeutic activities by modulating intracellular pathways (e.g., *miR-125b* inhibitors via stem cell-derived extracellular vesicles) or using receptor agonists (e.g., S1P). **(C)** Modulating surface molecules: Targeting surface components (e.g., chondroitinase affecting CS GAGs and MCK-2 interactions) can impact cellular communication and processes like pathogen entry. **(D)** Enhancing phagocytosis: Blocking "don't eat me" signals to promote uptake of undesirable cells (e.g., targeting CD47-SIRPα with anti-CD47 antibodies). **(E)** Modulating cytokine secretion: Controlling inflammatory or regulatory mediator release (e.g., blocking CCL22 to influence CCR4^+^ T cells) to shape immune responses. **(F)** Mφ delivery: Introducing therapeutic Mφ populations to disease sites (e.g., M2-polarized Mφs from PBMCs). **(G)** Genetic and pharmacological preservation of Mφs. Transient Hedgehog signaling activation (Ad-Shh/Ad-Gli1) regenerates Mφs and re-establishes homeostatic paracrine interactions (HGF, C1q). S1P pretreatment mitigates radiation-induced ROS and DNA damage in Mφs, restoring crucial growth factor secretion. Abbreviations: CS GAGs, chondroitin sulfate glycosaminoglycans; EV, extracellular vesicles; MCK-2, Mφ chemokine 2; Mo, monocytes; PBMCs, peripheral blood mononuclear cells; S1P, Sphingosine-1-Phosphate. Image created with BioRender.com.

Mφ plasticity and diverse phenotypes are increasingly recognized as key regulators of inflammation, anti-tumor responses, tissue repair, and regeneration. This understanding is driving therapeutic strategies that target Mφ-associated molecules or involve Mφ transplantation (121–124). In the context of SGs, Mφs are critical for both homeostasis and disease pathogenesis. Local manipulation of SG Mφs in animal models (via percutaneous glandular injection and retroductal infusion) and the use of SG organoids for *in vitro* studies are advancing our mechanistic understanding. Furthermore, novel therapeutic approaches targeting SG diseases are emerging, utilizing delivery of Mφ-related genes and molecules (**Figures 4A–D**). **Figure 4** conceptualizes a multi-pronged therapeutic framework centered on manipulating macrophages in salivary gland diseases. These distinct strategies, which range from modulating macrophage numbers to fine-tuning their effector functions, represent a sophisticated toolkit for therapeutically reshaping the glandular microenvironment. This deepening appreciation for the Mφ subset equilibrium is catalyzing a paradigm shift in therapeutic design. The goal of next-generation Mφ-targeted therapies is evolving beyond broad depletion or monolithic polarization towards a more sophisticated objective: restoring homeostatic balance (125). This entails developing precision strategies that can differentially modulate these subsets. Such approaches may involve selectively preserving and rejuvenating the pro-regenerative long-lived resident populations (46) while simultaneously taming the pathogenic inflammation driven by their short-lived counterparts. This focus on restoring the ecosystem, rather than simply targeting a single cell type, holds immense promise for developing truly restorative therapies for SG disorders.

### 6.1 Immune regulation for inflammation and viral responses

A critical therapeutic goal in SG pathologies is to modulate Mφ activity to achieve balanced inflammatory responses, rather than simply promoting a shift from pro-inflammatory M1 to anti-inflammatory M2 phenotypes. Contemporary immunology recognizes inflammation as a spectrum process (126) where a self-limiting, acute inflammatory response, orchestrated by M1-like Mφs, is an indispensable prerequisite for efficient debris clearance and the initiation of tissue regeneration (127, 128). Following radiation injury, for example, an innate immune-driven inflammatory cascade is fundamentally necessary for subsequent intestinal regeneration (128). Successful Mφ regulatory strategies must be tailored to specific disease phases—maintaining appropriate inflammatory responses during acute stages while facilitating inflammation resolution and tissue repair during chronic phases.

Irradiated SG exemplify this paradigm. Radiation acutely suppresses local immune competence—reducing resident Mφs and ILCs—thereby compromising physiological inflammatory responses. When applied within a narrow early window, transient Hedgehog–Gli activation restores both cell types and re−establishes a pro−reparative paracrine milieu (including HGF, C1q, and CSF1/IL34), rather than broadly "dampening" inflammation (16, 54). By contrast, at chronic stages after irradiation or in persistent inflammatory diseases such as oral cGVHD and SjD, therapeutic priorities shift toward promoting resolution and preventing fibrosis through stage−specific tuning of Mφ–stromal and Mφ–lymphocyte circuits (39, 57).

Within this framework, targeting the Mφ-driven CCL22–CCR4 axis can modulate CCR4^+^ T−cell recruitment and IFN−γ production in SjD, thereby ameliorating glandular damage (39) (**Figure 4E**). Ductal or local delivery of extracellular vesicles can reprogram Mφs toward pro−resolving/pro−repair states and support epithelial–stromal repair, although efficacy is contingent on EV source, cargo, route, and timing (129–132) (**Figure 4B**). In fibrotic contexts such as oral cGVHD, targeting profibrotic Mφ–fibroblast interactions—for example, with Hsp47 siRNA—offers a plausible antifibrotic avenue (57, 58) (**Figure 4A**). Lp−PLA2 remains an exploratory biomarker for Sjögren's−associated lymphoma risk (133). Finally, modulating host glycosaminoglycans may limit CMV entry and spread in a cell−type−dependent manner, but these strategies are still nascent and are not Mφ−specific (134) (**Figure 4C**).

Notably, the evidence base is largely murine; successful translation will require stringent definition of therapeutic windows, dosing, and delivery strategies. Given oncogenic liabilities, Hedgehog activation should be transient and locally restricted, accompanied by rigorous preclinical safety evaluation (16, 54). Future research directions should prioritize elucidating the distinct functions of Mφ subpopulations across various disease stages and developing precision-targeted interventions for specific subsets within appropriate therapeutic windows.

### 6.2 Salivation restoration

Several regenerative strategies that leverage Mφ activity aim to restore SG function after damage. Therapeutic activation of the Hedgehog pathway, via gene delivery or small molecule agonists, has been shown to enhance resident Mφ function, promote angiogenesis, and ultimately rescue secretory function (16, 46, 54). Similarly, local administration of S1P protects resident Mφs and endothelial cells from radiation-induced damage by activating the S1pr1/Akt/eNOS pathway (47) (**Figure 4B, F, G**).

Another innovative approach is the intraglandular transplantation of conditioned M2-like E-MNCs, which restore function in radiation- and autoimmune-damaged glands by clearing cellular debris and secreting regenerative factors like IGF1 (102–104) (**Figure 4F**). A Japan clinical trial (jRCTb070190057) further underscores the translational relevance of this cell-based approach for radiation-induced salivary dysfunction (**Table 2**).

### 6.3 Tumor intervention

Autophagy impairment reprograms the tumor microenvironment by limiting Mφ and Treg infiltration. This effect, mediated by increased intratumoral glutamine, enhances CD8^+^ T cell infiltration and IFN-γ production, ultimately suppressing tumor growth. Dietary glutamine supplementation partially mimics this immunomodulatory effect (**Figure 3D**) (117).

Targeting TAMs is a promising strategy in solid tumors. Approaches include inhibiting recruitment/survival, depleting TAMs, polarizing pro-tumor phenotypes, and enhancing anti-tumor activity, including phagocytosis. Regulating TAMs can promote direct tumor cell phagocytosis and activate adaptive immunity (124, 135).

Key strategies targeting TAMs:

- Inhibition of recruitment/survival: Targeting the CSF1/CSF1R axis with drugs like pexidartinib, emactuzumab, and cabiralizumab (124) (**Figure 4A**).
- Enhanced phagocytosis: A key strategy to reinvigorate anti-tumor immunity is the therapeutic blockade of the CD47-SIRPα checkpoint. Cancer cells frequently upregulate surface CD47, which ligates the inhibitory receptor SIRPα on Mφs to suppress phagocytosis. Agents such as magrolimab, a monoclonal antibody against CD47, physically obstruct this ligand-receptor interaction. This effectively unmasks the malignant cells, unleashing Mφ-mediated phagocytic clearance (**Figure 4D**).
- Other methods: Trabectedin (used for sarcomas) may deplete TAMs.

It is important to emphasize that the clinical translation of Mφ-targeted therapies for SG diseases remains at an early and exploratory stage, with limited direct clinical evidence available to date. A review of clinical trial databases reveals that the current, limited clinical evidence is derived from broader solid tumor cohorts that include patients with SG cancer, reflecting the challenges posed by the rarity and heterogeneity of these malignancies. Some researchers have explored TAM-related targets, primarily CD47 blocking antibodies and CSF1R inhibitors. These strategies aim to modulate the tumor microenvironment by enhancing Mφ phagocytosis or reducing TAM survival. Notably, a US trial (NCT02216409) used anti-CD47 antibodies Hu5F9-G4 in advanced cancers, including those of the SG and head and neck, to enhance Mφ-mediated tumor cell elimination (136). Similarly, CSF1R inhibitors have been explored in this context to suppress TAM survival; a Taiwan trial (NCT02734433) investigated pexidartinib in submandibular pleomorphic adenocarcinoma and adenoid cystic carcinoma (137, 138), and a US trial (NCT02526017) evaluated cabiralizumab with cabozantinib in head and neck solid tumors. A China trial (NCT06048367) investigated the iron-loaded carbon nanoparticle CNSI-Fe(II) in advanced solid tumors, including head and neck cancers. This agent utilizes a dual mechanism: it directly induces tumor cell ferroptosis and reprograms TAMs toward an anti-tumor M1 phenotype via iron-mediated oxidative stress (**Table 2**). These clinical trials offer promising directions for Mφ-targeted therapies in SG tumors.

The efficacy of anti-CD47 immunotherapy in SG tumors depends on CD47 expression within the tumor microenvironment, emphasizing the need for histology-driven approaches (116). Further studies on CD47 expression across subtypes are crucial to determine therapeutic efficacy. The heterogeneity and complexity of TAMs pose significant challenges for targeted therapies. Integrating next-generation sequencing and molecular characterization could provide insights for personalized TAM-targeted therapies. While some trials (e.g., magrolimab) faced safety concerns in hematological malignancies, they are still being evaluated in solid tumors. Combination therapies, including immune checkpoint inhibitors, show promise. Chimeric antigen receptor (CAR) Mφ (CAR-M) represent an emerging immunotherapeutic approach for solid tumors, but their potential in SG tumors is unexplored.

SG tumors present unique challenges due to their histological diversity, anatomical location, and distinct tumor microenvironments. The application of Mφ-targeted therapies to SG disorders remains largely theoretical, with most evidence derived from preclinical models (112). Future clinical trials specifically focusing on SG pathologies are needed to establish the efficacy of Mφ-targeted approaches in these contexts.

### 6.4 Biomaterial-assisted targeted drug and cell delivery

The efficacy of Mφ-directed therapies can be significantly enhanced by advanced delivery systems. Biomaterials offer a powerful platform for improving the retention and phenotypic modulation of therapeutic Mφs or for delivering drugs directly to Mφs *in situ* (123, 135, 139). Emerging technologies include Mφ-biomimetic nanoplatforms that leverage the natural inflammatory tropism of Mφs for targeted delivery, hydrogel-based delivery systems, and DNA nanomaterials that can be taken up by Mφs to regulate their function (140, 141). For instance, retroductal delivery of drug-loaded apoptotic extracellular vesicles (ApoEVs) has shown potential in restoring Mφ autophagy and function in age-related SG dysfunction (84). This field remains nascent for SG diseases but holds immense promise for increasing the precision and effectiveness of future Mφ-centric therapies.

To conclude, targeting SG Mφs offers promising therapeutic strategies for salivation restoration, tumor intervention, and inflammation modulation. Mφ-targeted therapeutic strategies for SG disorders are evolving toward precision modulation of specific subsets and functions. Promising interventions include transient pathway activation to preserve resident Mφs, delivery of conditioned Mφs to promote tissue repair, and targeted inhibition of pathogenic signaling axes. Emerging delivery technologies, including biomaterials and extracellular vesicles, offer improved therapeutic precision and efficacy. Clinical translation of Mφ-targeted therapies for SG diseases remains at an early stage, with limited disease-specific clinical trials. Optimal therapeutic windows for intervention, particularly the timing of pro-inflammatory versus pro-resolution approaches, are incompletely defined. Predictive biomarkers for patient stratification and treatment response monitoring are lacking. The long-term safety of Mφ modulation strategies requires rigorous evaluation. Development of salivary biomarkers reflecting Mφ activation states represents an untapped opportunity for non-invasive disease monitoring.

## 7 Conclusion and perspectives

The study of SG Mφs challenges conventional views of glandular immune homeostasis, revealing novel non-immune functions in processes like secretion and tissue repair, and thus provides a powerful paradigm for Mφ biology across exocrine and mucosal tissues. These roles are orchestrated through an intricate interplay with the microenvironment encompassing epithelial, neural, and vascular cells. A central tenet emerging from this review is that SG health hinges on a dynamic equilibrium between long-lived, resident "guardian" and short-lived, monocyte-derived "sentinel" Mφ populations. While this conceptual framework offers a unifying lens through which to view glandular physiology and pathology, it is crucial to acknowledge its current frontiers. Direct evidence delineating this balance within the SG remains nascent, with many insights extrapolated from other organ systems. This limitation, however, defines a critical and exciting research trajectory: to elucidate the precise molecular and cellular mechanisms that establish, maintain, and ultimately disrupt this equilibrium in the unique SG microenvironment. Unraveling these mechanisms is paramount for developing the next generation of truly restorative therapies—interventions aimed not at simple Mφ ablation, but at the sophisticated restoration of their homeostatic balance.

Despite increasing recognition of SG Mφ functions, frontiers remain:

Unravelling mechanistic depth and cross-disciplinary impacts: Future research must define how microenvironmental cues dictate Mφ dynamics and effector functions, including immunometabolic reprogramming and complex neuro-epithelial-immune networks, further exploring how sexual dimorphism in macrophage ontogeny interacts with age-related functional declines, such as impaired autophagy, to create a microenvironment permissive for autoimmune diseases like SjD. Precisely identifying sources of key regulatory factors like CSF1 and validating inferred immunoregulatory functions *in vivo* are paramount. Comparative analyses across SGs and other tissues will further illuminate their unique contributions and conserved mechanisms, offering new insights into Mφ biology, tissue microenvironment interactions, and glandular physiology. Such findings will provide models of neuro-immune-epithelial cell interactions that extend research beyond the oral domain.

**Figure 5:**
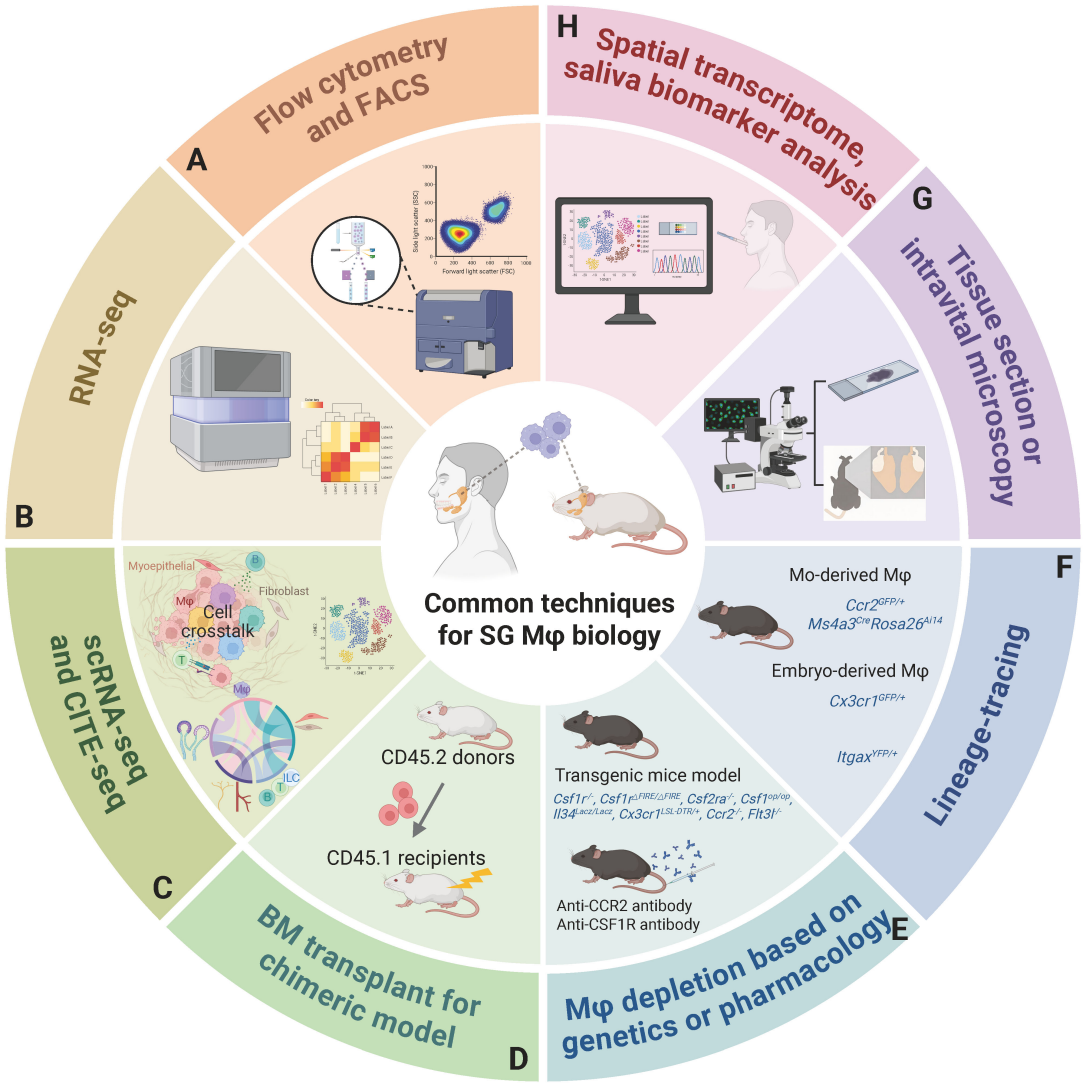
A comprehensive overview of methodologies to investigate SG Mφs. **(A)** Flow cytometry and FACS for cell identification, quantification, and isolation. **(B)** RNA sequencing offers a global view of SG Mφs' gene expression, revealing their functional states in health and disease. **(C)** ScRNA-seq, including CITE-seq for simultaneous protein profiling, enables high-resolution analysis of Mφ heterogeneity and interactions. **(D)** Bone marrow transplantation for chimeric models providing a controlled in vivo system to study the origin and development of SG Mφs. **(E)** Genetic (using transgenic models) or pharmacological Mφ depletion strategies are employed to remove SG Mφs and assess their impact on SG physiology and pathology, providing functional validation. **(F)** Lineage tracing utilizes specific genetic markers (e.g., distinguishing monocyte-derived and embryo-derived Mφs) to track the origins, developmental trajectories, distribution, and functional differences of distinct SG Mφ populations. **(G)** Tissue section microscopy visualizes SG morphology and Mφ localization, while intravital microscopy provides real-time, dynamic observation of SG Mφ behavior. **(H)** Future directions: Spatial transcriptomics for spatial gene expression analysis and Mφ relationships, and saliva biomarker analysis for non-invasive assessment of SG and systemic health. Abbreviations: CITE-seq, cellular indexing of transcriptomes and epitopes by sequencing; FACS, fluorescence-activated cell sorting; scRNA-seq, single-cell RNA sequencing. Image created with BioRender.com.

Harnessing advanced methodologies: Integrating single-cell and spatial multi-omics, high-dimensional flow cytometry, *in vivo* and *ex vivo* imaging (e.g., whole-tissue transparency, real-time imaging, 3D reconstructions), and organoids will precisely map subgroup heterogeneity, spatial localization, and intricate cell-cell interactions. Developing subtype-specific Mφ depletion and gene delivery tools for preclinical models like sialolithiasis, and critically, for classical oral diseases like dental caries and periodontitis, is also vital (**Figure 5**).

Bridging murine models and human disease for translational considerations: While murine models have provided an indispensable framework for defining the core tenets of SG Mφ biology—including their CSF1R-dependency, resident CX3CR1^+^ populations, and niche-specialized subsets at epithelial and neurovascular interfaces—a formidable translational gap separates these fundamental discoveries from clinical reality. Closing this divide necessitates a critical synthesis of human and murine data to delineate conserved mechanisms, acknowledge profound species-specific differences, and strategically address key knowledge gaps.

Preliminary key conservation patterns across species have emerged: Comparative analyses by Zhao et al. of mouse submandibular and human parotid gland single-cell datasets have confirmed three major SG resident Mφ subsets with conserved transcriptional signatures across species. Despite these similarities, significant translational challenges persist: Murine SG Mφs exhibit a striking sexual dimorphism in their ontogeny and maintenance. This phenomenon remains entirely unexplored in humans but may hold the key to understanding the pathophysiology of female-predominant disorders such as SjD.

The reliability of cross-species comparisons is further undermined by systematic data biases and a paucity of clinically relevant human data. A tripartite "species-gland-disease" bias pervades the literature: murine research is dominated by studies of the submandibular gland, whereas human analyses are skewed towards clinically accessible tissues like the parotid (from tumor resections) or minor labial glands (from SjD biopsies). Moreover, critical human single-cell atlases are conspicuously absent for key pathological and developmental states—including post-radiation injury, obstructive disease, and embryogenesis—which prevents direct validation of mechanisms elucidated in corresponding mouse models.

These foundational disparities create significant hurdles for translating therapeutic strategies. For instance, transient Hedgehog pathway activation, a promising pro-regenerative strategy in mice and swines, faces an uncertain path to the clinic due to concerns over long-term oncogenic risk and an undefined therapeutic window in humans. The success of other Mφ-centric interventions, whether targeting chemokine axes or employing extracellular vesicles, will ultimately depend on rigorous validation of target expression and function within the unique immunological context of human SGs. Current Mφ-targeted therapies are predominantly tested in general solid tumor cohorts. Dedicated trials focusing on specific SG pathologies are essential to establish clinical efficacy in neoplastic and non-neoplastic disease contexts. Thus, the imperative for future research is clear: to construct human single-cell and spatial atlases directly comparable to preclinical models, to develop sophisticated humanized systems that recapitulate human disease, and to design biomarker-driven clinical trials that can finally bridge the chasm between basic discovery and patient benefit.

Ultimately, deciphering the molecular dialogue between SG Mφs and their niche will not only redefine our understanding of SG diseases but also offer broader insights into other glandular pathologies. Critically, this review will foster paradigm shifts in immune homeostasis and neuro-epithelial-immunology, thereby positioning salivary Mφ-related factors as non-invasive indicators of both oral and systemic immune health.
